# Supplementary material for: Roles of the interaction with children and families in mediating the association between digital health literacy and well-being of early childhood teachers in Portugal: A cross-sectional study
Source: PLoS One. 2023 Sep 20;18(9):e0291748. doi: 10.1371/journal.pone.0291748 (PMC10511109; doi:10.1371/journal.pone.0291748)
Supplement: S1 Table — (DOCX) [file pone.0291748.s001.docx]

Supplementar Table 1 – Associations between DHL and ECE teachers´ well-being and their interaction with children and families

|  | **DHL**  Information searching | **DHL**  Adding self generated content | **DHL**  Evaluating reliability | **DHL**  Determining relevance |
| --- | --- | --- | --- | --- |
|  | OR (95%, CI) | OR (95%, CI) | OR (95%, CI) | OR (95%, CI) |
| ECE teachers´ well-being (median) | **1,96 (1,36; 2,82)** | 0,80 (0,46; 1,37) | 1,16 (0,84; 1,59) | **1,48 (1.00; 2.19)** |
| Adult-child interactions (median) | **1,63 (1,14; 2,32)** | 1,13 (0,67; 1.93) | **1,46 (1,06; 2,00)** | **1,54 (1,05; 2,26)** |
| Emotional climate (median) | 1,41 (0,99; 2.00) | 0,86 (0,50; 1,46) | 1,09 (0,80; 1,49) | 1.02 (0,70; 1,49) |
| Interaction with families (median) | **2.02 (1,41; 2,89)** | 1,14 (0,67; 1.93) | **1,59 (1,15; 2,18)** | **1,95 (1,32; 2,89)** |

Bold: p<0.05

Binary regression models having ECE teachers´ Well-being (median split) and interactions and emotional climate as outcomes and DHL dimensions as predictors.
