# Supplementary material for: Roles of the interaction with children and families in mediating the association between digital health literacy and well-being of early childhood teachers in Portugal: A cross-sectional study
Source: PLoS One. 2023 Sep 20;18(9):e0291748. doi: 10.1371/journal.pone.0291748 (PMC10511109; doi:10.1371/journal.pone.0291748)
Supplement: S2 Table — (DOCX) [file pone.0291748.s002.docx]

Supplementar Table 2 Associations between ECE teacher´s interaction with children, families and emotional climate, and well-being

|  | Adult-child interactions  OR (95% CI) | Emotional climate  OR (95% CI) | Interaction with families  OR (95% CI) |
| --- | --- | --- | --- |
| ECE teachers´ Well-being (median) | **1.43 (1.24; 1.65)** | **1.40 (1.24; 1.59)** | **1.24 (1.10; 1.40)** |

Bold: p<0.05

Binary regression models having ECE teachers´ Well-being (median split) as outcome and interactions and emotional climate as predictors.
