# Supplementary material for: Roles of the interaction with children and families in mediating the association between digital health literacy and well-being of early childhood teachers in Portugal: A cross-sectional study
Source: PLoS One. 2023 Sep 20;18(9):e0291748. doi: 10.1371/journal.pone.0291748 (PMC10511109; doi:10.1371/journal.pone.0291748)
Supplement: Attachment — Submitted filename: Response_reviewers.docx [file pone.0291748.s003.docx]

**Response to the Editor:**

1.  Please ensure that your manuscript meets PLOS ONE's style requirements, including those for file naming.

***Authors #1***

*We thank the editor, we updated the style requirements. Please see the revised version of the manuscript.*

2-Please change "female” or "male" to "woman” or "man" as appropriate, when used as a noun

***Authors #2***

*We thank the editor, we updated the manuscript according with the suggestion.*

3-We note that the grant information you provided in the ‘Funding Information’ and ‘Financial Disclosure’ sections do not match.

***Authors #3***

*We thank the editor, we updated the manuscript according with the suggestion.*

4- Data Availability statement.

***Authors #4***

*We thank the editor,* all DHL descriptive files are available from the

https://doi.org/10.34622/datarepositorium/LF7URM

5- Please include your tables as part of your main manuscript and remove the individual files.

***Authors #5***

*We thank the editor, we included the tables and figure into the main manuscript and removed the individual files.*

6- Please include captions for your Supporting Information files at the end of your manuscript, and update any in-text citations to match accordingly.

***Authors #6***

*We thank the editor, we included the captions for our Supporting Information files at the end of your manuscript.*

7- Please review your reference list to ensure that it is complete and correct.

***Authors #7***

*The references are complete and correct.*

**Response to the Reviewers:**

**Reviewer #1:**

The author has clearly written the following points to support the manuscript.

1. Sampling technique

2. Data analysis

3. Linkage to conclusion

***Authors #1***

*We thank the reviewer for this reinforcement.*

________________________

The author has used binary logistic regression and mediation analysis that suits to the research topic to research desired results. The data has been collected mostly from 853 respondents which has been clearly stated.

The author is requested to read through the manuscript to get proper connectivity between the sub-topics used.

***Authors #2***

*We thank the reviewer for addressing this issue. We have included a proper connectivity between the binary logistic regression and mediation analysis. Please see the revised discussion of the manuscript. It now reads:*

*“Hence, ECE teachers may be better equipped to access and apply health information, enhancing interactions with children, and ultimately improving their well-being.” (lines 37-39.)*

*“Figure 1 shows the direct effect of DHL on ECE teachers´ well-being, mediated by ECE teacher-child interaction (indirect effect). Our findings indicate that the effect of DHL dimensions on well-being was partially mediated by ECE teacher-child interaction” (lines 190-193).*

**Reviewer #2:**

That is a good article but I think it needs English edition.

***Authors #1***

*We thank the reviewer for pointing the need of English edition. We updated the language throughout the manuscript. Please see the revised version of the manuscript.*

_________________________

The Introduction is so weak. Please discuss more about necessity, previous study. It is also proposed to present the findings of studies that are closer to the research topic and have pointed out the link between digital health literacy and educational environments.

***Authors #2***

*We thank the reviewer for pointing this. We discussed more about the need and findings from other studies, please see lines 38-42 and 45-52; it now reads: “Although health literacy has been criticized for neglecting the wider societal and structural dynamics that shape the individuals´ choices (1), contemporary health literacy explanations acknowledge that individuals´ skills are largely influenced by the environmental demands and complexities they face (2)”*

*“During an epidemic, an overwhelming amount of information may be generated, leading to an "infodemic" (3). To navigate these digital contexts, new competencies are required. In response to misinformation, strategies for infodemic management (4) are essential to address the challenges faced by communities during health emergencies. This has highlighted the importance of developing digital health literacy (DHL) skills (5) to enable individuals to access, understand, and apply health information in their daily lives in the digital age. Consequently, DHL has gained increasing attention from the academic community (6).”*

__________________

In the method, ethical considerations should be mentioned with more detail

***Authors #3***

*We thank the reviewer for pointing this out. Ethical considerations were detailed, please lines 102-106. It now reads “The study implemented appropriate organizational and technical measures to safeguard the collected data and minimize inherent risks associated with data processing. Such measures included pseudonymization, encryption, and access control. The collected data were used for the purposes of the research, and individual results were not disclosed or communicated to any third party.”*

__________________

In the discussion section, please provide more studies related to the research topic and compare the findings of the current research with other related studies.

***Authors #4***

*We thank the reviewer for pointing this out. We included more studies related to the research topic and compare the findings of the current research with other related studies. Please see the revised version of the manuscript. It now reads:*

*“There is a growing body of literature that has found associations between interactive health literacy and various health outcomes (7-11). This suggests that individuals who are able to effectively understand and engage in shared decision-making are more likely to have better health outcomes. Also, there is a growing body of evidence highlighting schools as critical settings for improving health literacy. (12, 13). Research has shown that schools can play a crucial role in improving health literacy, not only for students but also for teachers and staff. Health literacy education in schools can provide individuals with the knowledge and skills necessary to make informed decisions about their health and well-being (12-14).” (lines 339-347).*

_____________________

The strengths and weaknesses of the study should be presented at the end of the discussion.

***Authors #5***

*We thank the reviewer for pointing this out. The limitations and strengths of the study were presented at the end of the discussion.*

________________________

Some references need to be updated.

***Authors #6***

*Some references were updated, please see the revised version of the manuscript.*

_________________________

**References**

1.	The Lancet. Why is health literacy failing so many? Lancet (London, England). 2022;400(10364).

2.	Nutbeam D, Muscat D. Health Promotion Glossary 2021. Health promotion international. 2021;36(6).

3.	Zarocostas J. How to fight an infodemic. Lancet. 2020;395(10225):676.

4.	World Health Organization. WHO competency framework: building a response workforce to manage infodemics. Geneva: World Health Organization; 2021.

5.	van der Vaart R, Drossaert C. Development of the Digital Health Literacy Instrument: Measuring a Broad Spectrum of Health 1.0 and Health 2.0 Skills. J Med Internet Res. 2017;19(1):e27.

6.	Yang K, Hu Y, Qi H. Digital Health Literacy: Bibliometric Analysis. Journal of medical Internet research. 2022;24(7).

7.	Pelikan J, Ganahl K, Roethlin F. Health literacy as a determinant, mediator and/or moderator of health: empirical models using the European Health Literacy Survey dataset. Global health promotion. 2018.

8.	Brørs G, Dalen H, Allore H, Deaton C, Fridlund B, Norman C, et al. The association of electronic health literacy with behavioural and psychological coronary artery disease risk factors in patients after percutaneous coronary intervention: a 12-month follow-up study. European heart journal Digital health. 2023;4(2).

9.	Brørs G, Dalen H, Allore H, Deaton C, Fridlund B, Osborne R, et al. Health Literacy and Risk Factors for Coronary Artery Disease (From the CONCARD PCI Study). The American journal of cardiology. 2022.

10.	Wang L, Fang H, Xia Q, Liu X, Chen Y, Zhou P, et al. Health literacy and exercise-focused interventions on clinical measurements in Chinese diabetes patients: A cluster randomized controlled trial. EClinicalMedicine. 2019;17:100211.

11.	Hirooka N, Kusano T, Kinoshita S, Aoyagi R, Saito K, Nakamoto H. Association between health literacy and purpose in life and life satisfaction among health management specialists: a cross-sectional study. Scientific reports. 2022;12(1).

12.	Paakkari L, Balch-Crystal E, Manu M, Ruotsalainen J, Salminen J, Ulvinen E, et al. Health-literacy education drives empowerment and agency. Lancet (London, England). 2023;401(10374).

13.	Okan O, Paakkari L, Jourdan D, Barnekow V, Weber M. The urgent need to address health literacy in schools. Lancet (London, England). 2023;401(10374).

14.	Nutbeam D. Health literacy as a public goal: a challenge for contemporary health education and communication strategies into the 21st century. Health Promot Int. 2000;15(3):259-67.
