# Supplementary material for: Roles of the interaction with children and families in mediating the association between digital health literacy and well-being of early childhood teachers in Portugal: A cross-sectional study
Source: PLoS One. 2023 Sep 20;18(9):e0291748. doi: 10.1371/journal.pone.0291748 (PMC10511109; doi:10.1371/journal.pone.0291748)
Supplement: Attachment — Submitted filename: Response to the Reviewersjuly.docx [file pone.0291748.s004.docx]

**Response to the Reviewer:**

**Reviewer #2:**

I would like to thank you for this research.

In general, the manuscript is written with an easy-to-follow and readable layout, the abstract is good and the problem is well-defined.

***Authors #1***

*We thank the reviewer for this reinforcement.*

________________________

The Study Area is clearly and appropriately defined. However, the manuscript needs some modifications including:

Please show your study type in the title (Place, time and type of study)

**Authors #2**

*We appreciate the reviewer's suggestion. The title of the study has been revised to: "Roles of the interaction with children and families in mediating the association between digital health literacy and well-being of early childhood teachers in Portugal: a cross-sectional study."*

________________________

The keywords should be set according to the Mesh term.

**Authors #3**

*We appreciate the reviewer's feedback. The keywords have been revised to align with MeSH terms. The revised version of the manuscript now includes the following keywords: school teachers, health, health literacy, infant, child, preschool, and family.*

________________________

Were all the teachers of 2908 institutions invited to the study?

**Authors #4**

*All the teachers from the 2 908 institutions contacted by e-mail, were invited to participate in the online survey using the platform SurveyMonkey. This information was clarified in the methods section. It now reads: “Overall, a total of 2 908 institutions were contacted by e-mail inviting all teachers to participate in the online survey using the platform SurveyMonkey” (lines 300-301)*

________________________

Refer to the table numbers in the text of the article.

**Authors #5**

*We included the table numbers in the text of the manuscript. We apologize for this oversight.*

________________________

A thorough revision of the language is needed to enhance the readability of the text.

**Authors #6**

*We have thoroughly revised the manuscript according to the comments and suggestions provided. Please see the revised version of the manuscript.*
